# Interactive Acupuncture for Poststroke Dysphagia: Protocol for a Randomized Controlled Trial

**DOI:** 10.2196/102785

**Published:** 2026-10-29

**Authors:** Zhiheng Zhao, Junjie Li, Manlin Zhao, Changjun Shao, Mingjie Xu, Yintao Zhang, Yueqiao Ren, Zhen Zhou, Fangfang Gao

**Affiliations:** 1 Department of Acupuncture and Encephalopathy, Second Affiliated Hospital of Tianjin University of Traditional Chinese Medicine, 69 Zengchan Road, Hebei District, Tianjin, 300250, China; 2 Graduate School, Tianjin University of Traditional Chinese Medicine, Tianjin, China; 3 Department of Dermatology, First Teaching Hospital of Tianjin University of Traditional Chinese Medicine, Tianjin, China; 4 National Clinical Research Center for Chinese Medicine, Tianjin, China; 5 School of Acupuncture and Moxibustion, Tianjin University of Traditional Chinese Medicine, Tianjin, China; 6 Department of Integrative Medicine, Tianjin Cancer Hospital Airport Hospital, Tianjin, China; 7 Department of Tuina and Rehabilitation, Hengshui Hospital of Traditional Chinese Medicine, No. 445 Shengli West Road, Taocheng District, Hengshui, Hebei, 053000, China, +86 18330853833

**Keywords:** poststroke dysphagia, four acupoints in the nape, interactive acupuncture, randomized controlled trial, swallowing function

## Abstract

**Background:**

Poststroke dysphagia (difficulty swallowing) is a common disabling complication of stroke and is associated with malnutrition, aspiration pneumonia, impaired quality of life, and mortality. Conventional swallowing rehabilitation and instrumental assessment are useful but may be limited by equipment dependence and inconsistent accessibility. Interactive acupuncture is a dynamic acupuncture procedure in which short needling stimulation at selected nape acupoints is synchronized with active swallowing tasks.

**Objective:**

This protocol describes a multicenter, parallel-group randomized controlled trial that will evaluate whether interactive acupuncture improves swallowing function in patients with poststroke dysphagia compared with conventional acupuncture at the same acupoints.

**Methods:**

A total of 100 participants with poststroke dysphagia will be randomized 1:1 to an interactive acupuncture group or a conventional acupuncture control group. Both groups will receive routine conventional medical care and standardized acupuncture therapy for stroke. The intervention course comprises 14 sessions delivered 6 times per week followed by a 1-month follow-up. The registered primary outcome is the water swallowing test (WST) grade; the primary efficacy comparison is the change in WST grade from baseline to immediately after the 14th treatment session. Secondary outcomes include the Standardized Swallowing Assessment, Functional Oral Intake Scale, Swallowing Quality of Life Questionnaire, instrumental and physiological assessments, inflammatory markers, safety outcomes, and a supportive WST responder analysis. Outcomes will be assessed at baseline; after sessions 3, 7, and 14; and at 1 month after treatment completion, where applicable.

**Results:**

The current amended protocol was approved by the Medical Ethics Committee of Hengshui Hospital of Traditional Chinese Medicine on August 25, 2026 (approval 2026-KY-09), and separate institutional ethics approval was obtained from the Second Affiliated Hospital of Tianjin University of Traditional Chinese Medicine (approval Y2025-053-01). The study was funded in June 2025. The trial was registered with the International Traditional Medicine Clinical Trial Registry on December 30, 2025. Recruitment began on January 1, 2026; as of July 6, 2026, a total of 36 participants had been enrolled. Primary outcome data analysis has not yet begun. Recruitment is expected to continue until June 1, 2027, with final follow-up anticipated by July 2027 and submission of the primary results planned for September 2027.

**Conclusions:**

This trial will clarify whether adding a task-synchronized interactive acupuncture procedure to the same acupoint prescription provides incremental benefit over conventional acupuncture for poststroke dysphagia and will provide a manualized, fidelity-monitored protocol for future clinical implementation.

## Introduction

### Background

Stroke is a leading cause of death and disability worldwide, and the burden of stroke-related disability remains substantial in China [1,2]. Poststroke dysphagia is one of the most clinically important sequelae because it is associated with malnutrition, dehydration, aspiration pneumonia, reduced quality of life, prolonged hospitalization, and increased mortality [3,4].

Current dysphagia management after stroke includes early screening, compensatory strategies, behavioral swallowing therapy, diet modification, and instrumental assessment when indicated [5-7]. Treatment selection and delivery should be individualized according to swallowing physiology, aspiration risk, and clinical status [3,5]. In practice, implementation may still be constrained by patient cooperation, equipment availability, institutional resources, and variable response in patients with persistent swallowing dysfunction [3,7].

In traditional Chinese medicine, poststroke dysphagia is commonly interpreted as impaired regulation of channel qi and obstruction of the throat orifice after stroke [8]. A recent multicenter randomized controlled trial suggested that acupuncture may improve swallowing function in patients with poststroke dysphagia [9]. Updated evidence syntheses also indicate potential benefit, although certainty is limited by the methodological quality and heterogeneity of the included trials [10,11]. A recent scoping review identified substantial variation in acupoint selection, stimulation mode, intervention dose, and reporting quality [12]. Experimental and clinical mechanistic studies suggest that acupuncture may influence swallowing-related cortical, brain stem, and peripheral sensory-motor pathways [13-15].

### Comparator Selection

The comparator is conventional acupuncture at the same 4 nape acupoints delivered via standard needle insertion, *deqi*, and 30-minute retention. It is not intended as a positive control in the pharmacological sense of a treatment with established efficacy used to demonstrate assay sensitivity. Rather, it serves as a standard reference treatment that represents current routine acupuncture practice for poststroke dysphagia in Chinese rehabilitation settings [16]. Against this reference, the trial estimates the incremental effect of adding the interactive manipulation component, and the comparison is therefore framed as a pragmatic superiority evaluation of two clinically implementable procedures rather than a mechanistic isolation of a single sensory element. This choice is consistent with the revised pragmatic-explanatory continuum indicator summary (PRECIS-2) framework, which emphasizes alignment between trial design decisions and the intended use of the results and recognizes usual care as an appropriate comparator in pragmatic trials [17].

Because the experimental and reference procedures differ in retention time and patient task engagement, performance-related differences may arise. This is a deliberate consequence of the design: task engagement and the timing of stimulation are constituent elements of the interactive intervention as it would be delivered in practice, and the trial evaluates the intervention as a whole rather than an isolated procedural element. In PRECIS-2 terms, these features relate to the domain of flexibility (delivery) because they define how the intervention is implemented under clinically feasible conditions rather than under an idealized mechanistic design. To limit bias, outcome assessors, data managers, and statisticians will remain blinded; allocation will be concealed before assignment; and intervention fidelity will be monitored using prespecified checklists in both arms.

### Study Rationale and Objectives

Interactive acupuncture is conceptualized as a task-synchronized needling procedure that combines local nape acupoint stimulation with active swallowing-related movement. The proposed mechanism is that time-locked peripheral stimulation and task performance may enhance sensory-motor integration in swallowing networks. This trial will evaluate the clinical effectiveness and safety of interactive acupuncture and explore related functional and objective indicators.

## Methods

### Trial Design

This is a prospective, multicenter, parallel-group randomized controlled trial with a 1:1 allocation ratio. A total of 100 participants will be enrolled after eligibility screening, baseline assessment, and written informed consent. Participants will receive 14 treatment sessions over approximately 2 to 3 weeks followed by a 1-month follow-up. The protocol follows the SPIRIT (Standard Protocol Items: Recommendations for Interventional Trials) 2025 recommendations for clinical trial protocols, and the intervention description was strengthened according to the TIDieR (Template for Intervention Description and Replication) principles [18,19].

The primary time point is after the 14th treatment session. Additional assessments will be performed after sessions 3 and 7 and at 1 month after treatment completion to describe the trajectory and durability of response.

### Trial Setting

The trial will be conducted at Hengshui Hospital of Traditional Chinese Medicine in Hebei Province and the Second Affiliated Hospital of Tianjin University of Traditional Chinese Medicine. The study timeline and trial flowchart are shown in Table 1 and Figure 1, respectively.

**Table 1.** Study timeline summarizing participant screening, informed consent, baseline assessment and randomization, representative treatment sessions, outcome assessments, safety monitoring, concomitant care, protocol deviation assessment, and follow-up. Session 1 is the first treatment session and is not an efficacy assessment time point. The primary water swallowing test (WST) comparison is from baseline to immediately after session 14; assessments after sessions 3 and 7 describe the treatment trajectory, and 1-month follow-up assessments are performed where applicable.

Item	Screening period (–7 to 0 d)	Baseline—visit 0 (session 0—baseline and randomization)	Treatment period				Follow-up period (visit 4—1 mo ±3 d after session 14)
			Session 1	Visit 1—session 3	Visit 2—session 7	Visit 3—session 14	
Enrollment							
Obtain informed consent	✓						
Inclusion and exclusion criteria	✓						
Baseline assessments		✓					
Randomization		✓					
Intervention and comparator							
Interactive acupuncture+basic treatment			✓	✓	✓	✓	
Conventional acupuncture+basic treatment			✓	✓	✓	✓	
Assessments							
WST		✓		✓	✓	✓	✓
SSA^a^		✓		✓	✓	✓	✓
FOIS^b^		✓		✓	✓	✓	✓
SWAL-QOL^c^		✓				✓	✓
VFSS^d^ and PAS^e^		✓				✓	
sEMG^f^		✓		✓	✓	✓	✓
Infrared thermography		✓		✓	✓	✓	✓
Inflammatory markers (IL-6^g^, TNF^h^, and CRP^i^)		✓				✓	
Safety							
Adverse events and safety evaluation		✓	✓	✓	✓	✓	✓
Concomitant medication or intervention		✓	✓	✓	✓	✓	✓
Protocol deviation assessment		✓	✓	✓	✓	✓	✓

a SSA: Standardized Swallowing Assessment.

b FOIS: Functional Oral Intake Scale.

c SWAL-QOL: Swallowing Quality of Life Questionnaire.

d VFSS: videofluoroscopic swallowing study.

e PAS: Penetration-Aspiration Scale.

f sEMG: surface electromyography.

g IL-6: interleukin-6.

h TNF: tumor necrosis factor.

i CRP: C-reactive protein.

**Figure 1.**
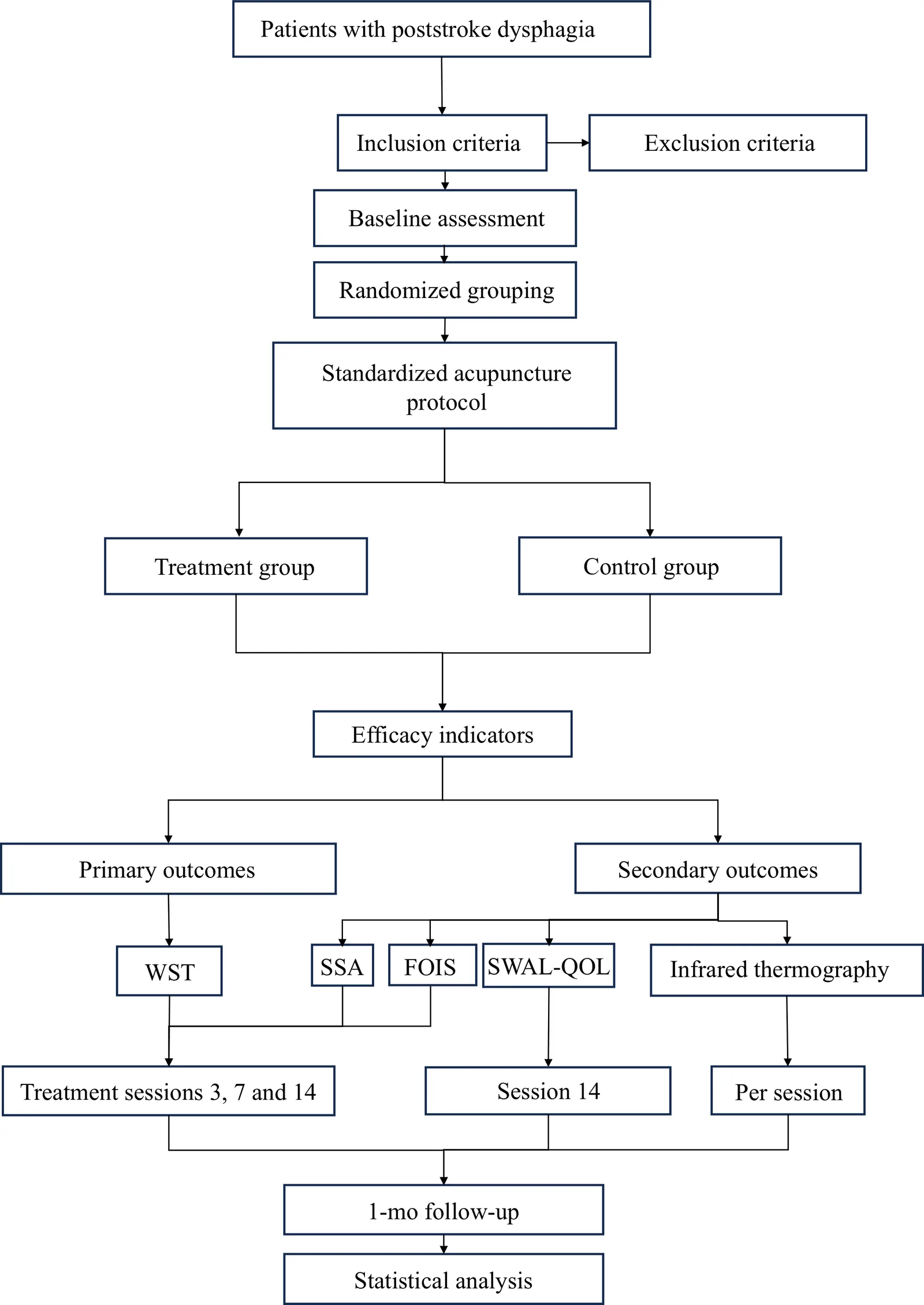
Trial flowchart. Eligible participants with poststroke dysphagia undergo eligibility screening, informed consent, and baseline assessment before 1:1 randomization to interactive acupuncture or conventional acupuncture. Both groups receive the prespecified basic treatment and complete 14 treatment sessions. Outcome assessments are performed at baseline; after sessions 3, 7, and 14; and at the 1-month follow-up where applicable, followed by prespecified statistical analysis. FOIS: Functional Oral Intake Scale; SSA: Standardized Swallowing Assessment; SWAL-QOL: Swallowing Quality of Life Questionnaire; WST: water swallowing test.

### Patient and Public Involvement

During the design phase, structured interviews and questionnaire surveys were conducted with patients with poststroke dysphagia and their family members. Their feedback informed the operational procedure, the timing of assessments, and approaches to improving acceptability and adherence.

### Participants

#### Eligibility and Exclusion Criteria

Eligibility and exclusion criteria are shown in Textbox 1.

Textbox 1. **Inclusion and exclusion criteria for participants with poststroke dysphagia. Eligibility is assessed before enrollment and randomization.**

**Inclusion criteria**

- Diagnosis of stroke with dysphagia according to clinical and swallowing assessment criteria
- Water swallowing test grade 3-5
- Convalescent phase of stroke, defined as 2 weeks to 6 months after onset, with stable vital signs and clinical condition
- Age of 18-75 years, any sex
- Ability to provide written informed consent personally or through a legally authorized representative when decisional capacity is insufficient

**Exclusion criteria**

- Severe cognitive or psychiatric impairment preventing cooperation with assessment or treatment, including Mini-Mental State Examination score <10
- Dysphagia caused by head or neck tumor, surgery, radiotherapy, structural disease, or other nonstroke factors
- Severe cardiac, hepatic, renal, or other systemic disease; critical illness; or unstable vital signs
- Local infection, skin damage, or injury at the needling area of the neck or back
- History of acupuncture syncope or extreme fear of acupuncture that precludes protocol adherence
- Participation at the time of enrollment in another interventional clinical trial likely to interfere with the outcomes of this study

#### Recruitment and Consent

Participants will be recruited from outpatient and inpatient rehabilitation services at the participating hospitals through physician referral, posters, and hospital electronic advertisements. Screening will be performed by trained investigators according to the eligibility criteria.

Because survivors of stroke may have communication or cognitive impairment, capacity to provide informed consent will be assessed by the investigator through clinical judgment; review of cognitive status; and a teach-back process to confirm understanding of the study purpose, interventions, possible benefits and risks, voluntary participation, and withdrawal rights. Participants with sufficient capacity will sign the consent form personally. If a participant lacks sufficient decisional capacity but is otherwise eligible, consent will be obtained from a legally authorized representative, and the participant’s assent will be sought whenever feasible. Participants may withdraw consent for intervention and/or data collection at any time without affecting their routine care.

#### Withdrawal and Discontinuation Criteria

A participant may discontinue the assigned acupuncture intervention for any of the following reasons: personal choice to stop treatment; a clinician decision that continuation is not in the participant’s best interest; occurrence of a serious or intolerable adverse event judged related or possibly related to the intervention; clinically significant disease progression, neurological deterioration, or a new medical condition requiring treatment incompatible with the protocol; pregnancy; poor adherence, defined in advance as completion of fewer than 80% of the 14 scheduled sessions; or loss of the capacity to provide continued informed consent. Participants who discontinue the intervention will remain in the trial and will continue to be followed for all scheduled outcome and safety assessments whenever possible unless they explicitly withdraw consent for further data collection. The reason, date, and circumstances of each discontinuation will be recorded.

A participant has the right to withdraw from the trial at any time, for any reason, and without any effect on their subsequent medical care. Withdrawal may be initiated by the participant (or legal representative) or, in exceptional circumstances, by the investigator for safety reasons. At the time of withdrawal, the investigator will clarify the scope of the participant’s decision, distinguishing between withdrawal from further intervention only, withdrawal from further study visits and prospective data collection, and withdrawal of consent for the use of all data. Where permitted and agreed, participants who withdraw from further visits will be asked for permission to collect key outcome and safety data through routine clinical records or a final assessment.

Following the intention-to-treat principle, participants who discontinue the intervention or withdraw will not be replaced, and all randomized participants will be analyzed in the group to which they were allocated, consistent with the treatment policy estimand described in the Statistical Methods section. The number of participants who discontinue or withdraw, together with the reasons, will be summarized by group and reported in the participant flow diagram.

### Interventions

#### Basic Treatment

Both groups will receive basic treatment according to cerebrovascular disease prevention and treatment guidelines and local clinical practice, including management of blood pressure, glucose, lipids, cerebral circulation, and neurological support as clinically indicated [20]. Both groups will also receive standard stroke acupuncture at GV26, PC6, SP6, HT1, LU5, and BL40 [21].

#### Control Group

The control group will receive conventional acupuncture at the same 4 acupoints: GV14, Ex-HN24, GV16, and GV15. GV14 and Ex-HN24 will be needled perpendicularly; GV16 and GV15 will be needled with the tip slightly directed toward the chin. After *deqi*, needles will be retained for 30 minutes without additional manipulation or guided task-synchronized interactive tasks. The same precautions and adverse event recording procedures will be applied.

#### Treatment Group

The treatment group will receive acupuncture at the same acupoints as the control group. Before needling, the practitioner will confirm participant identity; the session number; any adverse events since the previous session; alertness; posture; oral hygiene readiness; and swallowing safety, including the absence of contraindications for drinking. A small volume of water will be prepared according to the patient’s swallowing safety status as judged by the clinician. The 4 nape acupoints will be located and needled as in the control group.

After the 4 nape acupoints have been needled, the practitioner will manipulate the needle handles using small-amplitude lifting-thrusting and twirling at approximately 30 cycles per minute with an even reinforcing-reducing technique while one assistant guides the participant through active swallowing tasks and monitors swallowing safety. According to the participant’s swallowing safety status, the interactive task will consist of repeated swallowing and/or small-volume water drinking attempts synchronized with the needling manipulation and verbal cues. If coughing, choking, marked discomfort, dizziness, or another safety concern occurs, the interactive task will be stopped, and the participant will be reassessed. After 2 to 3 minutes of interactive stimulation at the 4 acupoints, the needles will be slowly withdrawn; no needle retention is used in this group. Treatment will be administered once daily 6 times per week for a total of 14 sessions.

#### Acupoint Location and Insertion Technique

Acupoint locations and corresponding insertion needling depth are shown in Table 2 and Figure 2.

**Table 2.** Acupoint locations and needle insertion techniques. The table summarizes the anatomical locations and planned insertion depth and direction for the standard stroke acupuncture points and the 4 nape acupoints used in both randomized groups.

Acupoint	Acupoint location	Needling depth
Shuigou (GV26)	On the face, at the junction of the upper third and middle third of the philtrum groove	Oblique insertion toward the nasal septum, 0.3‐0.5 cun^a^
Neiguan (PC6)	On the anterior forearm, 2 cun proximal to the distal wrist crease, between the tendons of palmaris longus and flexor carpi radialis	Perpendicular insertion, 0.5‐1 cun
Sanyinjiao (SP6)	On the medial aspect of the lower leg, 3 cun superior to the medial malleolus, on the posterior border of the tibia	Perpendicular insertion, 1‐1.5 cun
Jiquan (HT1)	In the center of the axillary fossa, at the pulsation site of the axillary artery	Perpendicular insertion, 1‐1.5 cun
Chize (LU5)	On the anterior aspect of the elbow, at the cubital crease, in the depression on the radial side of the biceps brachii tendon	Perpendicular insertion, 0.5‐0.8 cun
Weizhong (BL40)	On the posterior aspect of the knee, at the midpoint of the popliteal crease	Perpendicular or oblique insertion outward, 1‐1.5 cun
Dazhui (GV14)	On the posterior neck, in the depression below the spinous process of the seventh cervical vertebra, on the posterior midline	1.2‐1.5 cun
Chonggu (Ex-HN24)	Extra point on the neck, in the depression below the spinous process of the sixth cervical vertebra, on the posterior midline	Within 1.2 cun
Fengfu (GV16)	On the posterior neck, directly below the external occipital protuberance, in the depression between the trapezius muscles	Within 1.2 cun
Yamen (GV15)	On the posterior neck, at the spinous process of the second cervical vertebra, on the posterior midline	Within 1.2 cun

a One *cun* is a relative unit of measurement in acupuncture that differs from patient to patient; it is defined as the width of the thumb at the interphalangeal joint (approximately 25 mm in standard acupuncture needle measurements).

**Figure 2.**
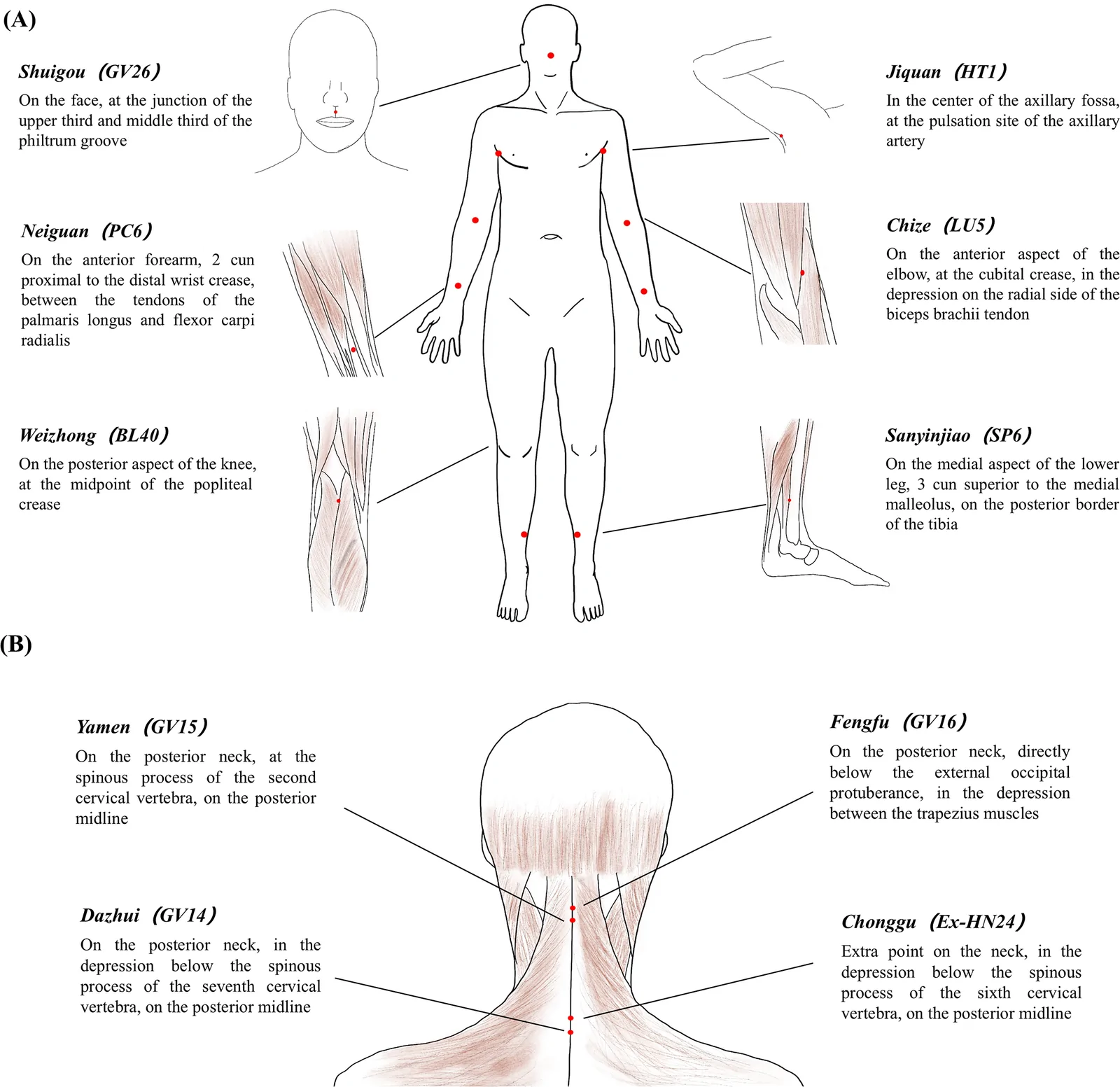
Locations of acupuncture points used in the trial: (A) the 4 nape acupoints used in both randomized groups: *dazhui* (GV14), *chonggu* (Ex-HN24), *fengfu* (GV16), and *yamen* (GV15) and (B) the standard stroke acupuncture points received by both groups: *shuigou* (GV26), *neiguan* (PC6), *sanyinjiao* (SP6), *jiquan* (HT1), *chize* (LU5), and *weizhong* (BL40).

#### Practitioner Training

All practitioners will be licensed traditional Chinese medicine physicians or acupuncturists with clinical acupuncture experience. Before trial participation, they will complete a standardized training program covering the rationale of the trial, acupoint localization, needling depth and direction, safety precautions for dysphagia, coordination of task-synchronized swallowing activities, adverse event management, documentation requirements, and blinding procedures. Competency will be assessed using a practical examination and an intervention checklist; practitioners must demonstrate correct acupoint localization, safe needle handling, correct sequence of interactive stimulation, and appropriate response to coughing or distress before treating study participants.

Fidelity will be monitored through session checklists completed at every treatment, periodic direct observation by the principal investigator or delegated monitor, review of case report forms, and cross-center meetings. Checklist items will include acupoint confirmation, needle depth and direction, presence or absence of retention, duration of interactive stimulation, completion of swallowing task guidance, adverse events, and protocol deviations.

### Protocol Deviations

Protocol deviations will be classified as minor or major before database lock. Database lock refers to the formal finalization of the trial database for the final statistical analysis after completion of data entry, data cleaning, and query resolution, after which no further routine data changes are permitted. Minor deviations include brief scheduling variation, incomplete checklist fields that can be resolved from source documents, or small deviations in treatment timing without expected impact on safety or the primary outcome. Major deviations include incorrect allocation, use of nonassigned intervention, failure to receive the allocated intervention course sufficiently for per-protocol inclusion, unblinding of outcome assessors, enrollment despite clear ineligibility, or concurrent treatment in another interventional trial likely to affect swallowing outcomes. All deviations will be documented with date, reason, corrective action, and assessment of impact. Major deviations will be reviewed by the principal investigator and considered in sensitivity and per-protocol analyses.

### Outcomes

#### Primary Outcome

The primary outcome is the water swallowing test (WST) grade, assessed before treatment (baseline) and after the 3rd, 7th, and 14th treatment sessions. The primary efficacy comparison is the change in WST grade from baseline to immediately after the 14th treatment session; the assessments after the 3rd and 7th sessions are prespecified repeated measurements used to characterize the time course of response. The WST is a 5-grade bedside swallowing assessment in which a lower grade indicates better swallowing function (grade 1=drinking water smoothly once without choking; grade 2=drinking water in more than one attempt without choking; grade 3=drinking water once with choking; grade 4=drinking water in more than one attempt with choking; grade 5=frequent choking or inability to complete the test [22]).

#### Secondary Outcomes

Secondary outcomes include supportive WST assessments after sessions 3 and 7 and at the 1-month follow-up; the overall WST response rate at session 14, defined as the proportion of participants with an improvement of at least 1 WST grade from baseline without worsening of aspiration-related clinical symptoms; Standardized Swallowing Assessment (SSA) score [23,24]; Functional Oral Intake Scale (FOIS) score [25]; Swallowing Quality of Life Questionnaire (SWAL-QOL) score [26]; videofluoroscopic swallowing study (VFSS) findings, including penetration and aspiration evaluated using the Penetration-Aspiration Scale where applicable [27]; surface electromyography (sEMG) parameters; infrared thermography of the pharyngolaryngeal region; and peripheral inflammatory markers, including interleukin-6, tumor necrosis factor α, and C-reactive protein. The WST, SSA, FOIS, VFSS, sEMG, infrared thermography, and inflammatory markers are clinician- or instrument-assessed outcomes. The SWAL-QOL is participant reported; if a participant has communication difficulty, trained assessors will assist by reading standardized questions without interpretation, and caregiver assistance will be recorded. The SSA and FOIS will be assessed after treatment sessions 3, 7, and 14. The SWAL-QOL will be assessed after the 14th treatment session. Infrared thermography will be performed at each treatment session.

Data collection will take place in the participating hospitals in designated assessment rooms or radiology facilities for VFSS. Outcome assessors will be trained and blinded to group allocation. Source data will be recorded on paper case report forms and then entered into the electronic database.

### Safety

All adverse events during the study, including local pain, bleeding, hematoma, dizziness, syncope, choking, coughing, aspiration-related symptoms, or other unexpected events, will be documented. Serious adverse events will be reported to the ethics committee according to institutional requirements and managed promptly.

### Sample Size

The target sample size was determined before participant recruitment based on preliminary clinical data obtained during the trial development stage. For sample size planning, change in WST grade was treated numerically. The preliminary data suggested a mean grade improvement of 1.5 (SD 1.2) in the control group and 2.5 (SD 1.2) in the intervention group, corresponding to an anticipated between-group difference of 1.0. To provide external support for the plausibility of the control group estimate, we cite a published clinical study in which the mean WST score decreased from 4.17 (SD 0.91) before treatment to 2.50 (SD 1.14) after treatment, corresponding to an observed improvement of 1.67 [28]. This published study is used as external corroboration of the original control group assumption rather than as the source of the original preliminary data.

With the original assumptions, a 2-sided α of .05, power of 90%, and a 1:1 allocation ratio, the PASS (NCSS LLC) calculation required 32 participants per group. During the original protocol development stage, a Bonferroni adjustment was applied by dividing the 2-sided α of .05 by 4 planned WST assessment comparisons (adjusted α=.0125), increasing the calculated sample size to 42 participants per group. After allowing for 15% attrition, the target sample size was set at approximately 50 participants per group, or 100 participants in total. We acknowledge that this Bonferroni adjustment was conservative and is not required once session 14 is explicitly specified as the single primary efficacy comparison. It is reported here only to transparently describe how the original prerecruitment target sample size was derived. The primary hypothesis test will use a 2-sided α of .05, and the session 3 and session 7 assessments will be analyzed as supportive longitudinal measurements. No sample size re-estimation based on accumulating efficacy data or interim between-group comparisons has been performed during recruitment.

### Randomization and Blinding

Eligible participants will be randomly assigned to either group in a 1:1 ratio. The sequence will be generated by an independent statistician using block randomization. Allocation concealment will be maintained with sequentially numbered, opaque, sealed envelopes opened by an independent coordinator only after enrollment and completion of the baseline assessment. Treating practitioners cannot be blinded because of the nature of acupuncture procedures. Participants will be informed that 2 active acupuncture procedures are being compared but will not be told the investigators’ hypothesis regarding superiority. Outcome assessors, data managers, and statisticians will remain blinded to allocation until the primary analysis is completed.

### Analysis Population

The full analysis set will include all randomized participants and will follow the intention-to-treat principle. Participants will be analyzed according to their randomized group regardless of adherence or treatment discontinuation, and missing primary outcome data will be handled using prespecified missing data methods. The per-protocol set will include participants who complete at least 80% of assigned treatment sessions, have primary outcome data at session 14, and have no major protocol deviations judged to affect efficacy evaluation. The safety set will include all participants who receive at least one session of the assigned intervention or control procedure and will be analyzed according to the treatment actually received.

### Data Management and Monitoring

Study data will be collected using standardized case report forms. Two trained researchers will independently enter the data into a dedicated electronic database, followed by cross-checking and logical validation. Personal identifiers will be stored separately from research data. An independent quality monitoring team will review consent documentation, intervention fidelity, completeness of outcome data, adverse event reporting, and protocol deviations at the mid-enrollment stage and study completion.

### Statistical Methods

Baseline characteristics will be summarized by group but not subjected to significance testing for comparability. Continuous variables will be summarized using means and SDs or medians and IQRs, and categorical variables will be summarized using frequencies and percentages.

The primary efficacy comparison concerns the change in WST grade from baseline to session 14 in the full analysis set. Because the WST is ordinal, the primary model will use proportional odds ordinal regression to compare session 14 WST grades between randomized groups while adjusting for baseline WST grade, study center, and prespecified clinically relevant covariates such as age and time since stroke onset [29]. The treatment effect will be reported as a common odds ratio with a 95% CI. If the proportional odds assumption is not met, alternative ordinal models and a Mann-Whitney *U* test on change scores will be used as sensitivity analyses. The overall response rate is a supportive secondary analysis and will be compared using a chi-square test or Fisher exact test with effect estimates and 95% CIs.

Repeated postbaseline outcomes will be analyzed using mixed-effects models or generalized estimating equations including group, time, group-by-time interaction, baseline value, and study center. Continuous outcomes such as SSA, FOIS, SWAL-QOL, sEMG, thermography, and inflammatory markers will be analyzed using linear mixed models when assumptions are met and appropriate transformations or nonparametric methods otherwise. Binary or ordinal outcomes, including penetration and aspiration categories, will be analyzed using generalized linear mixed models. Adherence will be summarized as number and percentage of planned sessions completed and will be reported by group.

Missing outcome data will be described by group and reason. Multiple imputation will be considered for missing primary outcome data under a missing at random assumption; sensitivity analyses, including complete-case and conservative imputation scenarios, will be used to assess robustness [30]. All tests will be 2 sided. The primary outcome will use a 2-sided α level of .05, whereas secondary outcomes will be interpreted as supportive and exploratory.

### Ethical Considerations

The current amended protocol, informed consent form, and related study documents were reviewed and approved by the Medical Ethics Committee of Hengshui Hospital of Traditional Chinese Medicine on August 25, 2026 (approval 2026-KY-09; protocol version date: August 25, 2026). This approval includes the protocol amendment correcting the planned sample size from 99 to 100 participants (50 participants per group). The Second Affiliated Hospital of Tianjin University of Traditional Chinese Medicine, as the other participating study center, also provided institutional ethics approval for participation in this multicenter trial (approval Y2025-053-01). The study will be conducted in accordance with the Declaration of Helsinki, relevant Chinese laws and regulations, and the International Council for Harmonisation of Technical Requirements for Pharmaceuticals for Human Use E6 (R3) guideline for good clinical practice [31]. Written informed consent will be obtained from all participants or legally authorized representatives before enrollment.

## Results

The study was funded in June 2025. The trial was registered with the International Traditional Medicine Clinical Trial Registry on December 30, 2025 (ITMCTR2025002622). Recruitment began on January 1, 2026; as of July 6, 2026, a total of 36 participants had been enrolled. Primary outcome data analysis has not yet begun. Recruitment is expected to continue until June 1, 2027. The last participant follow-up is expected by July 2027, database cleaning and locking are planned for August 2027, and submission of the primary trial results is planned for September 2027.

## Discussion

### Expected Findings

The design emphasizes clinical feasibility, standardized intervention delivery, blinded outcome assessment, and multidimensional evaluation of swallowing function.

The trial responds to two limitations in the current acupuncture literature for poststroke dysphagia. First, recent randomized and evidence synthesis studies suggest potential benefit [9,10], but aspiration-focused studies and scoping reviews continue to identify low-certainty evidence, intervention heterogeneity, and incomplete reporting [11,12]. Second, prior studies often provide insufficient detail regarding practitioner training, intervention fidelity, and handling of deviations. The present protocol, therefore, includes a manualized intervention procedure, competency assessment, fidelity monitoring, and prespecified analysis populations.

The active comparator design allows for the evaluation of the incremental clinical value of interactive manipulation beyond the effects of acupoint selection and routine acupuncture contact. This choice may reduce assay sensitivity for placebo-related effects, but it is appropriate for a pragmatic comparison between two clinically applicable acupuncture strategies.

Mechanistically, the study is informed by an animal circuit study showing modulation of motor cortex inputs to brain stem swallowing pathways [13], clinical neuroimaging evidence of swallowing-related brain remodeling [14], and experimental evidence linking electroacupuncture-related peripheral sensory signaling with cortical regulation of swallowing [15]. The interactive procedure may combine peripheral acupoint stimulation with task-specific swallowing input, potentially facilitating sensory-motor integration. This hypothesis will require further mechanistic validation beyond the clinical outcomes of this trial.

### Limitations

The study has limitations. Participants and practitioners cannot be fully blinded. The sample size is powered for the primary swallowing outcome rather than mechanistic indicators. VFSS may not be repeated in every participant if safety or clinical feasibility prevents assessment. These limitations will be mitigated through blinded assessment, standardized data collection, and transparent reporting of adherence, missing data, and deviations.

### Dissemination Policy

The results will be disseminated through peer-reviewed publications, conference presentations, and updates to the trial registry. Authorship will follow standard authorship criteria.

### Conclusions

This randomized controlled trial will evaluate whether an interactive, task-synchronized acupuncture procedure provides additional benefit over conventional acupuncture at the same nape acupoints for poststroke dysphagia. The findings will provide comparative evidence on swallowing outcomes and safety, while the manualized intervention and fidelity-monitoring procedures may support replication in future studies.

## Supplementary material

10.2196/102785 Checklist 1 SPIRIT 2025 checklist.

## Abbreviations

FOIS: Functional Oral Intake Scale
PRECIS-2: revised pragmatic-explanatory continuum indicator summary
sEMG: surface electromyography
SPIRIT: Standard Protocol Items: Recommendations for Interventional Trials
SSA: Standardized Swallowing Assessment
SWAL-QOL: Swallowing Quality of Life Questionnaire
TIDieR: Template for Intervention Description and Replication
VFSS: videofluoroscopic swallowing study
WST: water swallowing test
